# That recognised risk factors can explain past and present international differences in breast cancer incidence: misconceptions 5

**DOI:** 10.1038/bjc.2012.134

**Published:** 2012-07-24

**Authors:** R N Hoover

**Affiliations:** 1 Division of Cancer Epidemiology and Genetics, National Cancer Institute, National Institutes of Health, Bethesda, MD, USA

## Abstract

Recent discussions on research priorities in the United States have revealed a widespread assumption that known risk factors entirely explain the historic international differences in rates of breast cancer. In fact, formal investigations of this question, both by modelling between-country differences and studies of migrants, indicate that an appreciable amount of the international differences in this disease remains unexplained. If this is not recognised, opportunities for research on breast cancer aetiology may be lost.

Misconceptions and ill-founded theories can arise in all areas of science. However, the apparent accessibility of many epidemiology findings and popular interest in the subject can lead to additional misunderstandings. The article below is the fifth in an occasional series of short editorials highlighting some current misinterpretations of epidemiological findings. Invited authors will be given wide scope in judging the prevalence of the misconception under discussion. We hope that this series will prove instructive to cancer researchers in other disciplines as well as to students of epidemiology. *Adrian L Harris* and *Leo Kinlen*

Since the pioneering work of Segi (1955) and Haenszel (1961), international differences in breast cancer incidence and mortality, together with the progressive change in risk over time among migrant populations towards that of their adopted countries, have been recognised as important aetiological clues. High-risk countries in the West have had rates four to six times those in several Asian countries (Segi *et al*, 1957; Doll and Muir, 1970), whereas the risk among migrant groups to America from Asia has risen over the course of two to three generations, to levels comparable to, or even greater than, US Whites (Haenszel and Kurihara, 1968; Ziegler *et al*, 1993), indicating that the international disparities are due to environmental or life-style, and not genetic, differences. More recently, in parallel with the ‘westernisation’ of certain Asian countries, the geographic differences in rates dropped from 4–6-fold to 2–3-fold (Curado *et al*, 2007). It was initially considered unlikely that the major international differences could be explained by differences in established risk factors, including age at menarche, parity, age at first birth, and breast feeding (MacMahon *et al*, 1973). Subsequently, the strongly positive correlations existing at the country level between dietary fat intake and breast cancer rates suggested that diet might make a substantial contribution to these international differences (Armstrong and Doll, 1975), but decades of analytical work have failed to establish any major causal link.

This might have been expected to renew interest in what is unexplained, but this has not happened. Indeed, there are signs in research and funding circles (although admittedly not so far in the literature) that many now assume that the international differences are adequately explained, particularly by reproductive factors. Exactly why this change in attitude has occurred is not clear, although the declines in the international variation may have appeared to reduce what needs to be explained, overlooking the relevance of past differences. Also, the impressive results of large collaborative studies of breast cancer risk factors (Collaborative Group on Hormonal Factors in Breast Cancer 2002 etc) have emphasised the amount of the disease for which they are responsible, perhaps distracting attention from studies specifically addressing international differences.

There have been several quantitative attempts to assess the impact of these risk factors on geographic differences in risk, either by modelling or by studies of migrant groups. In an early example of the former, applied to a Japanese population with a breast cancer rate five times lower than in the US, this difference was reduced to over 2.5 times in one model and 1.6 in another (Pike *et al*, 1983) by incorporating reproductive and anthropometric risk factors (Table 1). The value of the second estimate has been questioned, as it was obtained by including in the calculation an assumption that obesity was a positive risk factor for premenopausal and postmenopausal breast cancer, which is now known to be incorrect for premenopausal disease. An analysis in 1990 (Hsieh *et al*, 1990) of data from a large international study of breast cancer conducted in the 1960s (MacMahon *et al*, 1970) estimated the impact of established reproductive and anthropometric risk factors on the five-fold difference in risk between Boston and Tokyo, with the estimate after adjustment for these factors dropping to 4-fold in one model and 2.8-fold in another (Table 1). Most recently, the Shanghai Women’s Cohort was analysed using age-specific breast cancer rates for US Whites, giving an expected number 2.8 times that actually observed (Linos *et al*, 2008), which fell to 1.43 following control for the reproductive and anthropometric risk factors (Table 1).

Breast cancer in Asian-American migrants was the subject of a population-based case–control study of breast cancer in Hawaii, Los Angeles and San Francisco, conducted in 1983–87 (Ziegler *et al*, 1993; Wu *et al*, 1996), which was able to reproduce the five-fold risk gradient seen internationally, using a variable characterizing migration status (Table 1). Asians born in the East and migrating to the West were classified into four groups: those from rural areas in the East and residing in the West for <8 years, those from rural areas and residing in the West for 8+ years, those from urban areas in the East and residing in the West for <8 years, and those from urban areas and residing in the for West 8+ years. The odds ratios (ORs) of breast cancer for these four groups compared to Asian-Americans born and always living in the West were 0.20, 0.48. 0.67, and 0.75, respectively. When the relevant reproductive risk factors were controlled for, the ORs remained essentially unchanged, at 0.25, 0.48, 0.69, and 0.78. Some of this minimal effect of adjustment could reflect the well-known fact that migrants are not a representative sample of residents of their homelands. Thus, these women, particularly those migrating at a young age, had substantially lower proportions having their first birth under age 20 than women in their homelands. Separating the migrant groups from their country of origin in this way may have allowed the effects of other, albeit unknown, risk factors to become evident. More recently, a study of Chinese-American women born in China investigated the relationship between a measure of acculturation and mammographic breast density, a strong risk factor for breast cancer (Tseng *et al*, 2006). Overall, women with the highest level of acculturation were three times as likely to have more dense breasts as the least acculturated (Table 1). When potential reproductive, anthropometric, and nutritional risk confounding factors were controlled for, this declined to two-fold.

There are concerns about the robustness of any of the above methods to assess adequately the impact of controlling for recognised risk factors. The modelling exercises are based on assumptions about underlying baseline rates and relative risks for two very different populations (Ziegler *et al*, 2008). Both the modelling approach and the migrant studies deal with substantial differences for a large number of highly correlated aetiological variables, some of which may be unrecognised as yet but, by virtue of their correlation with the known factors, would be adjusted for as well when the known factors are. This may be less of an issue with the migrant studies, with their potential for uncoupling of some of the correlations, and might explain the tendency for greater differences remaining after control.

This issue aside, findings to date suggest that an appreciable amount of the international differences in breast cancer are not explained by known risk factors or mechanisms. With respect to mechanisms, attempts to find a hormonal basis for reproductive risk factors have on the whole been disappointing, although in the present context the report that the level of endogenous oestradiol was about one-third higher in US Whites than in rural Asian women held promise (Shimizu *et al*, 1990). However, a large pooling study indicated that a doubling of oestradiol levels is associated with only a 29% increase in risk (Key *et al*, 2002), implying that a 33% difference in oestradiol was likely to make only a small contribution to explaining international differences. Similarly, the Asian-American study found non-significant (less than 5%) differences in oestradiol concentrations between Asian-born and US-born women, with no consistent trends across the migrant groups (Falk *et al*, 2002).

It is important to recognise that known risk factors have not been found to explain the entirety of breast cancer epidemiology. To assume otherwise may limit the opportunities for aetiological research and preventive interventions.

## Figures and Tables

**Table 1:** Relative risk of breast cancer or mammographic breast densities in high- and low-risk populations

			**Relative risk**	
**Comparison**	**Outcome**	**Study**	**Age adjusted**	**Age and breast-cancer risk factor adjusted**
US Whites vs Asians	Breast cancer	Pike *et al* (1983)	5.0	1.6–2.5*
	Breast cancer	Hsieh *et al* (1990)	5.0	2.7–4.1*
	Breast cancer	Linos *et al* (2008)	2.8	1.4
				
Most acculturated vs least acculturated Asian-Americans	Breast cancer	Wu *et al* (1996)	5.0	4.0
	Increased Mammographic Densities	Tseng *et al* (2006)	3.1	2.0

*Range depending on differing model assumptions.
